# The Work-Room: Hints on Repairing

**Published:** 1912-06-15

**Authors:** 


### INSTITUTIONAL HOUSEKEEPING.

### The Work-room: Hints on Repairing.

To mend a worn or torn article securely and without spoiling its appearance is by no means an easy task, and the careful management of an institution may be judged to a great extent by the state of repair of the household linen.

The linen cupboards should be lined with paper; they should not be situated in a damp place; each shelf should be labelled with the name of the article it is to hold; and there should be a list of the contents of the cupboard on the door. Each article when mended should be placed at the bottom of the pile, so that all may have equal wear.

It saves much time if holes and tears have been drawn together before sending to the laundry, as washing and wringing will always make the holes larger. Usually the ward sister has charge of the linen for her ward, and after having it looked over she either sends the articles to be mended to the work-room or retains simple mending to be done in the ward. The sewing-room maid should be responsible for arranging the mending, for cutting suitable patches for holes so that they will match and fit, for seeing that there is suitable mending material for darning, and for planning out the best method of repairing each article. The work is piled up in heaps according to its kind, so that when the various assistants arrive all may be ready to hand and no time be wasted.

All mending should be inspected by the matron or housekeeper, so that any careless work may be detected. Untrained needlewomen think that any kind of cobbling will do for a worn article, whereas great precision is required.

Sheets are often torn at the corners through careless pegging out on the line; these can be mended with strong tape, machined or hemmed, making a mitred point at the corner, or the hem may be ripped off and a new one made. Thin places are best darned with flax or flourishing thread, this being stronger than darning cotton, and threads running in one direction only will be found sufficient to strengthen the weak spot.

### A Use for Worn Sheets.

Holes can be patched with the best parts of a worn sheet, which is to be preferred to new material. The patch should be three-quarters of an inch larger than the hole, and after tacking it on straight it can be machined in place. The worn material should be cut away evenly to make a neat patch, and machined or hemmed on the right side. If one end of a sheet is badly torn, a whole piece can be inserted. Sheets always wear first in the middle, and when this happens the sheet may be split and the ends or sides machined together; but these are, of course, not as comfortable as sheets with no seam in the centre. Sheets for children's beds may also be made from the best parts of old sheets. Old sheets may be made into mattress or pillow covers, to protect the ticking, or used for pillow-slips or bolster cases. If the holes are roughly patched they can be used for ironing sheets or dust sheets, or broad strips may be machined round and placed between the mattress and the bed, to preserve the mattress. Odd corners of sheets may be hemmed and used for bedroom crockery.

Blankets require careful mending, as they are expensive to renew. All thin places should be darned across with wool to match; holes should be patched with a square or triangular piece of the same quality as the blanket, cut in the same direction and herringboned flat over the hole without turning in the edges; the hole should be trimmed tidily and the edges herringboned to the blanket. If worn very thin in the centre, the blanket can be treated in the same way as a sheet—split open and the two sides machined or herringboned together, so that the thick part will cover the patient.

Old blankets may be patched and used as ironing blankets, or the better parts can be cut into cot or under blankets, or used for the lower part of the bed. Covers for various utensils—hot-water bottles or splints—may be economically made from the pieces. For polishing furniture or floors soft pieces of worn-out blankets are most useful.

### Housekeeping Queries.

### *The Use of a Chafing Dish.*

Sister Kathleen and Nurse S.—You would both find a *Chafing Dish* the very thing for preparing simple dishes or keeping food hot for members of the nursing staff who are compelled to dine later than the others. It will prove very advantageous for the night nurses' use in the ward kitchen. In fact, these handy appliances are simply invaluable, for they can be moved about anywhere and soon pay their initial cost by the saving of the fuel otherwise needed. The apparatus consists of (1) an easily regulated methylated-spirit lamp with stand; (2) a hot-water pan for use when slow cooking is needed; (3) the "blazer" or pan in which the ingredients are placed. When slow cooking is necessary for keeping food hot, for scrambled eggs or for ingredients likely to burn, put the hot-water pan under the "blazer." For quick cooking, such as frying omelets, bacon, kidneys, etc., remove the water pan and let the "blazer" come in direct contact with the flame. Burn good spirit; that of poor quality yields but a feeble heat. Avoid waste of spirit by at once covering the lamp with the little metal cap provided, and by carefully regulating the flame during cooking. Spirit may also be saved by having all ingredients ready prepared—*e.g.*, parsley, meat, fish, etc., chopped, cheese grated, and so forth, before lighting up. It is safer to stand the lamp-stand on a metal tray.

### To Our Readers.

These columns are open to queries and discussion touching all domestic matters. Matrons are invited to send us the result of any experiments they are making with a view to promoting the comfort of the staff. Those who are dissatisfied in regard to the quality of the goods supplied on contract can have samples tested by forwarding them to the Editor of "Institutional Housekeeping." Estimates given of the cost of boarding patients, nurses, and staff.

